# Variation in the outcomes of an ant-plant system: Fire and leaf fungus infection reduce benefits to plants with extrafloral nectaries

**DOI:** 10.1093/jis/14.1.84

**Published:** 2014-07-07

**Authors:** L. P. Pires, K. Del-Claro

**Affiliations:** Universidade Federal de Uberlândia, Laboratório de Ecologia Comportamental e de Interação, Instituto de Biologia, MG, Brazil

**Keywords:** arthropods, cerrado, fungus, mutualism, Qualea multiflora

## Abstract

Interactions between species are evolutionary malleable and may suffer changes in small timescales. Environmental disturbances, such as fire, can deeply affect species interactions, but how they influence the outcome of a mutualistic interaction has yet to be studied. In order to test the hypothesis that an environmental disturbance, in this case fire, may produce differences in the outcome of the association of ants with the extrafloral-nectaries-bearing plant *Qualea multiflora* Mart. (Myrtales: Vochysiaceae), a previous study was replicated, but this time after fire incidence, at the same study site and with the same plant species. Eight ant species visited *Q. multiflora* , and the most abundant genera were *Crematogaster* , *Cephalotes* , and *Camponotus* . Herbivores were found in branches with and without ants with no statistical difference, but foliar herbivory was always higher in branchs where ants were absent. Leaves were infested by fungi, and fungi spots were higher in branches where ants were present. Compared to the previous study, it was clearly observed that ant benefits to *Q. multiflora* varied over time. The most common ant species still protected leaves against chewing herbivores, but a new kind of leaf damage appeared, namely fungi spots. Data also support that ants may be acting as vectors of fungi spores on plants, as ant visited branches had higher fungus incidence than non-visited branches. Fire is a major source of disturbance in tropical savannas, and we suggest that it can cause strong variation in the outcomes of interactions between ants and plants with extrafloral nectaries in the Brazilian tropical savanna.

## Introduction

In ant-plant interactions, plants offer resources such as shelter (domatia, dead stems) and/or food (exudates, food bodies) to ants, who in return may protect the plants against herbivores, which they may prey upon or drive away ( Rico-Gray and Oliveira 2007 ). Extrafloral nectar (EFN) is the most common nutritional reward that plants offer to ants ( Koptur 1992 ), increasing survivorship, growth and reproduction of associated ant colonies ( Byk and Del-Claro 2011 ). EFN-producing glands have been present in Angiosperms since at least the Oligocene ( Pemberton 1992 ), attracting ants that eat, molest, or drive away herbivores, thus reducing leaf area loss ( Rutter and Rausher 2004 ) and/or increasing fruit ( Nascimento and Del-Claro 2010 ) and seed production ( Vesprini et al. 2003 ), with a positive impact on seed viability ( Sobrinho et al. 2002 ).

However, the effectiveness of ant services to plants may vary from positive to negative. For example, Nahas et al. (2012) showed complementary effects of multiple predators resulting in benefits to a plant possessing EFNs. Some authors pointed out a few ant partners as parasites ( Ness et al. 2006 ; Byk and Del-Claro 2010 ), and others failed to detect any benefit of the presence of ants to plants ( Rashbrook et al. 1992 ; Mody and Lisenmair 2004 ). Lack of ant protection can be explained by (1) differences in ant-foraging behavior among habitats ( Inouye and Taylor 1979 ), (2) variable susceptibility of distinct herbivore groups to ant predation ( Ito and Higashi 1991 ), (3) differences in the deterring skills among species of visiting ants ( Dejean et al. 2000 ; Byk and Del-Claro 2010 ; Nahas et al. 2012 ) or (4) difficulties in assessing seasonal variation and long-term outcomes in the ant-plant-herbivore interactions ( O’Dowd and Catchpole 1983 ; Del-Claro and Oliveira 2000 ). In the latter case, although habitat disturbance, for example fire ( Friese et al. 1997 ), is a cause of reduction in biodiversity ( Bond et al. 2005 ), environmental disturbances influencing the outcome of plant-ant interactions have yet to be studied.

Of the world’s biomes, ant-plant interactions are most frequent in tropical habitats, occurring in the full range of habitats where trees are found, from savannah to dense forest. These interactions are particularly pervasive in cerrado (Brazilian tropical savannah) due to the high incidence of insect- and plant-derived exudates on foliage, which promote intense ant activity on vegetation ( Oliveira and Freitas 2004 ). The cerrado biome of South America covers about 2 million km ^2^ , representing ca. 22% of the Brazilian land surface, and is the most diverse tropical savanna in the world ( Oliveira and Marquis 2002 ). Despite its great importance in area and diversity, few studies on interspecific associations have been conducted in cerrados ( Oliveira and Marquis 2002 ; Del-Claro and Torezan-Silingardi 2009 ).

In 1996, researchers performed an ant-exclusion experiment in a preserved cerrado reserve protected from fire occurrence, having as a model the tree *Qualea multiflora* Mart. (Myrtales: Vochysiaceae), which has extrafloral nectaries. The results showed that ants provide benefits to the plants, significantly reducing leaf area loss and increasing fruit set production ( Del-Claro et al. 1996 ). By the end of the dry season of 1999 (October) up to 2003, that cerrado reserve suffered incidental fire that completely burned the area in the dry season for three consecutive years (K. Del-Claro, personal observations). Because fire is such an important environmental factor in savannah vegetation ( Bond et al. 2005 ), it may influence the nature of tritrophic interactions between ants, herbivores, and plants with EFNs. Thus, our current study repeated the experiments of Del-Claro et al. (1996 in the same study site and with the same system after measures to stop fires were taken and the vegetation clearly recovered. The main aim of this study was to test the hypothesis that environmental disturbance, in this case fire, may produce significant differences in the outcomes (reduction of leaf area loss and fruit set production) of an ant-plant association.

## Materials and Methods

Field work was conducted from September 2008 to March 2009 in the cerrado *sensu stricto* ( Oliveira-Filho and Ratter 2002 ) ecological reserve (400 ha) of Clube de Caça e Pesca Itororó de Uberlândia (CCPIU) - MG, Brazil (18°59’S; 48°18’W). Fishing and hunting activities in the area have been prohibited since 1984. Experiments took place at the same area in CCPIU where Del-Claro et al. (1996) established their field experiments. The regional climate is markedly seasonal with a dry winter (April to September) and a rainy summer (October to March) (for additional details about the study site see Réu, Del-Claro 2005 ).

*Qualea multiflora* is an arboreal, decidual tree (Vochysiaceae) that possesses paired EFNs in leaf bases and floral pedicels. This species is common in the study site, at a density of 73 individual per hectare (see Del-Claro et al. 1996 for a detailed review). In September of 2008, trees (N = 19) of *Q. multiflora* of similar size (about 2 m high; similar number of branches and general aspect; phenological state: all of them without leaves and resprouting) were tagged with small plastic bands. Four branches similar in size and phenology (with resprouting gems) were selected in each plant. By the flip of a coin, two of these branches were designated as control and two as treatment. In control groups, branches did not receive any manipulation and ants had free access to them. In treatment groups, ants were all manually removed from the brances, and the intersection of the branch and the trunk was covered with a 3 cm large adhesive paper strap and a layer of sticky resin was applied over it (Tree Tanglefoot®) to prevent ants from climbing. All structures that could be used by ants as bridges to get access to these experimental branches were removed, and the integrity of the sticky barrier was checked weekly. To ensure that the sticky resin did not interfere with the results in control brances, the branch and trunk intersection was covered with a paper strap, and the resin was applied only on one side of the branch, allowing ants to climb freely.

In order to sample EFN-visiting ants and plant herbivores, branchs of both groups were checked fortnightly regarding number and species (or morpho species) of observed individuals. Voucher specimens were collected in non-experimental plants and deposited at the Museu de Biodiversidade do Cerrado of Universidade Federal de Uberlândia, Brazil.

Herbivory (leaf area loss) in the first month of experiments (September) was considered zero due to the fact that plants were resprouting and without leaves. It was checked again three and six months later. To determine mean herbivory per branch, data of nine leaves per branch were recorded, three from the most apical part, three from the middle, and three from the most basal part, near the intersection with the trunk. This procedure was done without leaf removal. Measurements of herbivory rates were assessed by placing leaves on a transparent grid (divided into millimeters). An index of herbivory from each leaf was calculated as the proportion of points in the grid falling within damaged and undamaged areas of the leaf blade (e.g., Korndörfer and Del-Claro 2006 ). A new type of leaf area loss was observed during field data collection: leaf spot/blight fungus infection. This was also recorded. Additionally, the number of floral buds and fruits produced by each branch was counted.

The data on leaf herbivory per plant (%) was arcsine converted to achieve normality, and a repeated measures ANOVA (with a post comparison Fishers’ test) was performed to compare the mean percentage of herbivory between branches with and without ants over time (December and March). We used Mann-Whitney *U* -test to assess if the presence or absence of ants influenced the amount of herbivores in treatment and control branches. The Mann-Whitney *U* -test was also used to verify which of the groups (treatment or control) showed the higher percentages of foliar fungus spots. All statistical analyses were performed following Zar (1999) .

## Results

**Figure 1.**
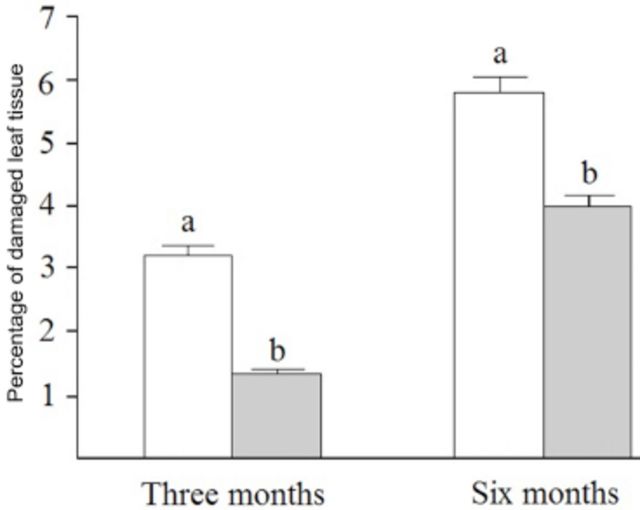
Leaf area loss (herbivory in %) between branches of *Qualea multiflora* (N = 19 tress) without (treatment - white bars) and with (control - grey bars) extrafloral-nectar-visiting ants three and six months after the beginning of experiments. Different letters point out statistical differences (repeated measures ANOVA, *p* <0.001). High quality figures are available online.

A total of eight ant species visited the EFNs of experimental plants, with the predominance of *Crematogaster* , *Camponotus* and *Cephalotes* genera ( Table 1 ). All ant species visited all control plants, and in several cases more than two distinct species could be found simultaneously in the same plant. There was no difference in the abundance of herbivores (chewing and/or sucking) in branches with (13.71 ± 8.06; *x* ± SE) and without (15.00 ± 8.09; *x* ± SE) ants ( *U* = 166; *P* = 0.879, Mann-Whitney *U* -Test). However, the foliar herbivory differed between branchs visited or not by ants ( *F*_1,17_ = 654.924; *P* < 0.01, repeated measures ANOVA). Branches without ants showed a higher herbivory in both sample periods ( Figure 1 ).

**Table 1.**
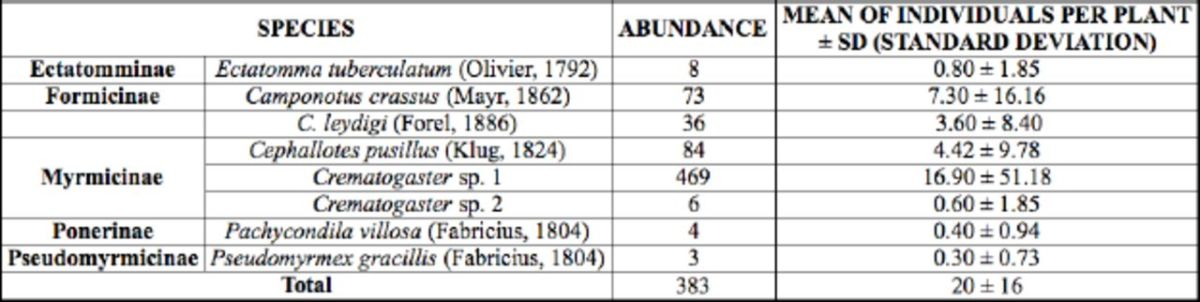
Ant species visiting extrafloral nectaries of *Qualea multiflora* in the cerrado savanna vegetation of Uberlândia, MG, Brazil (September 2008 up to March 2009).

On the other hand, leaf fungus abundance was significantly higher ( *U* = 113, *P* < 0.05; Mann-Whitney *U* -Test) in branches visited by ants (39.47 ± 2.59; *x* ± SD) than in branches not visited by ants (32.28 ± 2.205; *x* ± SD). Plants produced few buds and flowers, and there was no difference in fruit production between control and treatment branches.

## Discussion

Interactions between species are evolutionary malleable and may suffer spatial-temporal changes even in small timescales in response to biotic and abiotic factors ( Thompson 1999 , 2012). Variations over time in meteorological and biotic (mainly species composition) conditions may directly influence the outcomes of ant-plant-herbivore sybranchs (e.g., Rosumek et al. 2009 ). The results of our study support these hypotheses. Despite the maintenance of ant protection against chewing herbivores (reducing leaf area loss) over time (e.g., Del-Claro et al. 1996 ), a new type of loss in photo-synthetic leaf area appeared after fire occurrence: fungul infection, spots that covered, in some cases, almost the whole leaf (>90% of leaf area). It is important to say that one cannot assert that fungus occurrence was caused by fire, it just appeared after it. Why did this fungus emerge only after fire? Very little is known about the effects of fire on endophytic and parasitic fungi biota (see McMullan-Fischer et al. 2011 for a review), but some authors suggest that there may be an increase in fungi diversity and abundance following stress caused by fire ( Friese et al. 1997 ), as symbiotic mycorrhizal fungi occurs in orchid species in Australia ( Brundrett 2007 , 2009).

In the *Q. multiflora* case, one might ask if visiting ants are directly associated to the spread of leaf fungus infection. It could be considered as a negative collateral effect of a mutualistic association. Data showed that branches visted by ants (control) were more infested with fungi than ant-excluded branchs. There are no published studies showing a direct influence of fire on fungul infection in plant leaves. This fungus could be an opportunistic infection transmitted by air ( Levetin and Dorsey 2006 ), the visit of some undetected sucking bug ( Stout et al. 2006 ), or it can also have ants as a vector ( Hölldobler and Wilson 1990 ). However, this hypothesis requires further investigation.

Abiotic factors, including light and nutrient level, are influenced by fire occurrence in the Brazilian tropical savanna and can modify the outcome of ant-plant interactions ( Kersch and Fonseca 2005 ; Del-Claro and Marquis 2012). Fire can be associated with reduction in fruit production in *Q. multiflora* trees. In cerrado, fire destroys mainly the apical meristem of trees, altering plant architecture and soil quality ( Kauffman et al. 1994 ; Bond and Midgley 2001 ). These factors isolated or in combination can be responsible for the reduction in bud, flower, and fruit production in the plants (Kaufmann et al. 1994; Medeiros and Miranda 2008 ), which prevented an investigation of the effects of ants on plant fitness, as previously observed ( Del-Claro et al. 1996 ). Additionally, the successive occurrence of fire between the years of 1999 and 2003 may have amplified the negative effects in this ant-plant interaction. Fire can reduce both ant biomass and diversity, potentially reducing the ants’ protective effect on plants ( Langevelde et al. 2003 ). However, neither ant diversity nor abundance reduction was observed after disturbance caused by fire. Ant species visiting *Q. multiflora* varied in abundance and diversity when comparing our data with that from Del-Claro et al. (1996) . However, the common species associated with EFNs in cerrado, such as *Camponotus, Ectatomma, Cephalotes,* and *Crematogaster,* were the same as previously observed and remained actively visiting *Q. multiflora* trees even after fire. Other studies demonstrated that these ants have also remained active in other plants in the same study site (e.g., Byk and Del-Claro 2010 ). Nests sheltered in thick trunks and roots can survive fire occurrence and quickly produce satellite nests in dead branchs of neighboring plants in Brazilian cerrado ( Yamamoto and Del-Claro 2008 ; Knoechelmann and Morais 2008 ).

A major fire-coping strategy among Cerrado woody plants is vigorous resprouting after fire ( Vieira et al. 1996 ; Otterstrom and Schwartz 2006 ). Following resprouting, cerrado plants are especially attractive to herbivores, not only due to the presence of softer tissues that have higher nitrogen content (e.g., Vieira et al. 1996 ), but also due to the fact that there are fewer food options for herbivores ( Price 1991 ). In the cerrado, young leaves of plants with EFNs will produce more nectar than old ones (Korndörfer and Del-Claro 1996). Thus, more nectar and more herbivores will also attract more ants to the plants, considering that the majority of ant partners on EFN-plants in cerrado are omnivorous, feeding primarily on meat (live arthropod prey) and nectar (e.g., Del-Claro and Oliveira 1993; Moreira and Del-Claro 2005 ; Sendoya et al. 2009 ). Thus, one can suppose that visiting ants are especially important as a biotic defense for plants with EFNs after fire occurrence.,

Another important difference between this study and that of Del-Claro et al. (1996) is that following the fungul infection, which reduced the leaf area available to herbivores by almost 40%, no difference in herbivore abundance was observed between control and ant-excluded branchs. Microorganisms that infest plants, such as fungi, can deeply mediate herbivore-host plant interactions ( Fisher et al. 1986 ; Bergelson and Lawton 1988 ). Endophytic fungi infection can negatively affect the performance and survival of herbivores on host plants ( Hammon and Faeth 1992 ). Fungal infection may also promote physical and nutritional changes in plant tissues, mainly by the production of mycotoxins ( Strongman et al. 1990 ), which make leaves less attractive to herbivores (e.g., Cheplick and Clay 1988). Futhermore, Oki et al. (2009) demonstrated that fungal infection is higher in older leaves, which may be an explanation for the nonsignificant slight increase in herbivory during the study period, since herbivores may avoid infected leaves. Thus, one might suspect that in continuance of fungul infection, the diversity of associated fauna to *Q. multiflora* tress will be reduced.

Ecological forces promote changes in the outcomes of mutualistic interactions, so that they may become conditional mutualisms (Del-Claro 2004). Environmental disturbances are examples of such forces and they can deeply affect community structure and species interactions in many ways (e.g., cascade effects) ( Marquis 2005 ). Fire is a major source of disturbance in tropical savannas ( Oliveira and Marquis 2002 , and references therein), and the data obtained in this study suggest that it can cause variation in the association of of ants and EFN-bearing plants in the Brazilian tropical savanna.
